# Primary diffuse large B-cell lymphoma of the bone: bendamustine and rituximab are able to overcome resistant disease

**DOI:** 10.1186/2193-1801-3-342

**Published:** 2014-07-07

**Authors:** Patrizia Mondello, Michael Mian, Carmela Arrigo, Vincenzo Pitini

**Affiliations:** Department of Medical Oncology, University of Messina, Messina, Italy; Department of Hematology & CTMO, Hospital of Bolzano, Bolzano, Italy; Department of Hematology & Oncology, Medical University of Innsbruck, Innsbruck, Austria

**Keywords:** Primary bone lymphoma, Relapse, Bendamustine

## Abstract

Primary bone lymphoma (PBL) is a rare disease for which specific therapeutic guidelines have not yet been established. Due to common appearance in the elderly and recurring relapses, new treatments are required. We report the case of multiple relapsed aggressive PBL effectively treated using Bendamustine and Rituximab. A 78-year-old male patient presented with a painful mass in the left arm. Computed tomography (CT) showed a pathological tissue in the humerus diaphysis infiltrating the muscle, confirmed by positron emission tomography (PET) scan. Indeed, PET excluded pathological local lymph node involvement. Biopsy of the humerus revealed the presence of diffuse large B cell lymphoma. Recommended treatments for PBL were used, but relapses after an initial complete response occurred. Following the positive experience of Vacirca et al. the patient underwent Bendamustin 90 mg/mq gg1-2 q28 plus Rituximab 375 mg/mq q28 (BR). Herein we report the first experience of BR combination in PBL and it proved to be an efficacious and safe salvage therapy in relapsed/refractory PBL.

## Background

Primary bone lymphoma (PBL) is a rare disease and accounts for approximately 5% of extranodal lymphomas (Mikhaeel 2012). Almost all lymphoma entities can appear as PBL (Zinzani et al. 2003), but up to 80% are diffuse large B-cell lymphomas (DLBCL) (Mikhaeel 2012; Jaffe 2009). Despite its relatively good short-term prognosis, some patients eventually relapse (Ford et al. 2007). Treatment options are very limited in this setting, especially for elderly patients who are not usually eligible for standard salvage regimens followed by autologous stem cell transplantation (ASCT). Bendamustine plus rituximab (BR) has proven to be effective and well tolerated in relapsed/refractory DLBCL (Vacirca et al. 2014; Ohmachi et al. 2013) and might therefore be an interesting alternative for elderly patients suffering from relapsed/refractory PBL.

Herein, we present the first reported case of relapsed/refractory PBL which was successfully treated with bendamustine in association with rituximab.

## Case description

**Figure 1:**
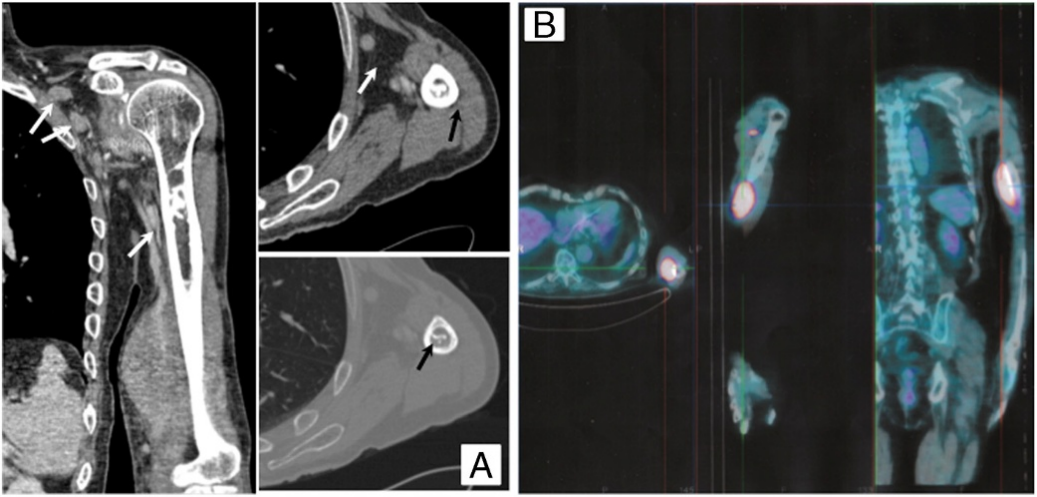
**Computed tomography (CT) and Positron emission tomography (PET) images at the time of diagnosis.** Panel **A**: CT scan showing the pathological tissue of the mid-diaphysis of the left humerus infiltrating the adjacent biceps brachii muscle for a longitudinal extent of 10.5 cm and axial dimensions of up to 5.5 cm. Several lymph nodes placed in the supraclavicular (1 cm), subclavian (2 cm) and in the axilla (19 mm). Panel **B**: PET image at the diagnosis with FDG uptake restricted to the primary mass, sparing locoregional lymph nodes.

**Figure 2:**
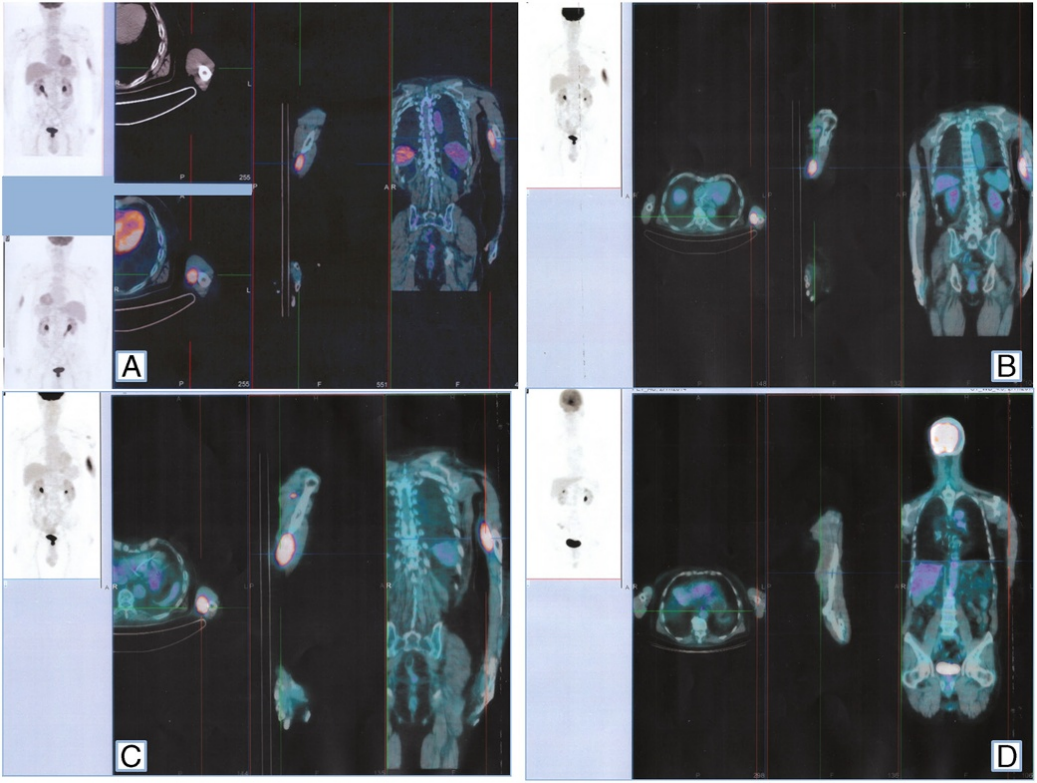
**PET image after second (Panel A), third (Panel B) and fourth relapse (Panel C) as well as after BR treatment (Panel D).**

A 78 year-old man with a left arm pain which had been worsening for 4 months was admitted in March 2008 to the orthopedic department. Physical examination revealed the presence of a large humeral mass and concomitant axillary lymphadenopathy. Computed tomography (CT) showed a pathological tissue in the humerus diaphysis infiltrating the muscle and dubious involvement of locoregional lymph nodes (Figure 1, Panel A). Positron emission tomography (PET) confirmed the presence of bone disease while the locoregional lymph nodes were not fluorodeoxyglucose (FDG) avid, (Figure 1, Panel B) suggesting a reactive lymphadenopathy. Biopsy of the humerus revealed the presence of a DLBCL. In immunohistochemistry, the neoplastic tissue was cluster of differentiation (CD) number 20 positive, CD79alfa +, CD 10+, CD3-, CD5-, CD23+, BCL2-, BCL6+ with a Ki67 of 30%. According to the algorithm by (Hans et al. 2004), it was sub-classified as germinal center B-cell–like DLBCL. Successive bone marrow trephine biopsy excluded the presence of bone marrow involvement. In accordance with the WHO classification for “Tumours of Soft Tissue and Bone”, this patient was affected by a primary bone lymphoma (Unni and Hogendoorn 2002). Because of muscle involvement *per continuitatem* a stage IV EA bulky was assigned. Due to age and stage, the International Prognostic Index (IPI) was 2. The patient was in a good clinical condition (performance status of 0) and echocardiography revealed a normal left cardiac function (Lang et al. 2005) with an ejection fraction of 70%. Therefore we administered in first line 6 cycles of rituximab, cyclophosphomide, doxorubicin, vincristine and prednisone (R-CHOP). Because CT scan showed only a partial remission (PR) and pain persisted, 90Y-ibritumomab tiuxetan (90Y-IT) consolidation was administered on the basis of the positive experience published by (Zinzani et al. 2008). Also in our case the radioimmuneconiugate was able to induce a PET-confirmed complete response (CR). One year later he suffered a painful, local, histological-proven relapse (Figure 2, Panel A). Due to the advanced age (79 years), poor performance status at the time of relapse and his ineligibility to ASCT, the patient underwent bimonthly administration of Rituximab for two years in order to spare as much toxicity as possible. After 2 months the patient’s general conditions improved and after 18 months he achieved a second CR. Since at this time the disease was confined to the bone without invasion of the surrounding structures, bisphosphonates were administered concomitantly. However, six months later, a second local relapse occurred, which (as with the previous ones) was proved by CT-PET (Figure 2, Panel B). Again no other tissues apart from the bone were involved so the patient underwent radiotherapy (RT) without any systemic treatment obtaining a CR. Only four months later he presented a painful mass in the left arm with bone and muscle involvement. Ultrasonography showed a patchy and hypoechoic area of 45x35 mm in the left biceps and X-ray revealed an osteolytic area of the humerus diaphysis. The suspicion of relapse was proven by biopsy and PET (Figure 2, Panel C) as well as by bone marrow trephine biopsy which excluded other disease localizations. Due to the lack of other treatment options, he finally underwent Bendamustine 90 mg/m^2^ days 1–2 q28 plus Rituximab 375 mg/m^2^ every 28 days for 6 cycles. Because of the advanced age, pegfilgrastim and antibiotic prophylaxis were administered to prevent neutropenia and infectious complications. The treatment was well tolerated. Reversible hematologic toxicity, mainly consisting of grade 2 neutropenia, occurred after the fourth cycle. Non extra-hematologic toxicities were registered with the exception of moderate fatigue. After the first cycle local pain regressed, after the second the mass was no longer palpable and after the fourth a PET-CT was carried out (Figure 2, Panel D) which confirmed CR so the remaining two cycles were administered. This result was quite unexpected since the patient had been heavily pretreated and the third relapse occurred only four months after RT. Moreover, the patient remained in CR for 12 months until he suffered the fourth relapse.

## Discussion

Because of the rarity of this extranodal lymphoma entity, no specific standard treatment has been established up to now. In the 1990’s PBL first-line treatment consisted of anthracycline containing therapy combined with RT (Dubey et al. 1997; Baar et al. 1994), nevertheless more recent data suggests that the latter can be omitted when administering at least 6 cycles of chemotherapy (Ramadan et al. 2007; Kim et al. 2012). The role of consolidation RT in limited-stage PBL is not well defined and remains a matter of debate. Indeed, in some retrospective studies (Phan et al. 2010; Beal et al. 2006) the addition of RT to an antracycline-based chemotherapy has demonstrated to improve the outcome, while in others it has not led to a reduction of relapses and survival rates when compared to patients who were treated only with chemotherapy (Haddy et al. 1988; Ramadan et al. 2007; Bruno Ventre et al. 2014). Moreover, only a few years ago, a large randomized trial of the GELA group showed that the addition of RT consolidation after CHOP chemotherapy in patients with localized stage DLBCL did not provide any advantage over CHOP alone (Bonnet et al. 2007). One year later, a German study confirmed in a historical comparison that the addition of RT to chemotherapy in patients with bulky and extranodal disease who achieved CR after R-CHOP14 did not improve PFS (Pfreundschuh et al. 2008a). However, all these studies were retrospective and the number of patients was relatively small, which is why a prospective confirmatory trial is needed. Since the addition of the monoclonal anti CD20 antibody rituximab significantly improved the outcome (Ramadan et al. 2007; Persky et al. 2008) and PFS of patients with localized DLBCL without leading to a significant increase of toxicity (Pfreundschuh et al. 2011, 2008b), it was administered concomitantely to CHOP in the present case as well.

Given that after induction treatment the patient achieved only a PR, consolidation treatment was needed. Due to the relatively low level of evidence for RT in this setting as mentioned above and considering the positive results of the phase II trial by (Zinzani et al. 2008), 90Y-IT consolidation was administered and the patient achieved a CR.

At the time of the first relapse, in order to positively influence the course of the disease, like in many other neopalsias (Coleman et al. 2012), bisphosphonates were added to the immunotherapy. Although the antineoplastic mechanisms of bisphosphonates are not fully clarified yet, their positive effect on disease control can be at least partially explained by alteration of the bone microenvironment (Power and Bird 2009; Guise 2008). Moreover, at this time the patient was in poor clinical conditions and due to his advanced age and inelegibility for a platinum containig salvage chemotherapy as well as successive autologous stem cell transplantation, we decided to administer bimonthly Rituximab in order to provide an efficient treatment without excessive toxicity (Coiffier et al. 1998; Rothe et al. 2004; Tobinai et al. 2004). Unexpectedly the patient achieved a second CR. Nevertheless, he relapsed again and since PBL was still confined to the initial site of the disease, he successfully underwent RT, in line with a previously published experience (Dubey et al. 1997).

At the third relapse, due to the lack of other treatment options and to the positive experience of (Vacirca et al. 2014)^,^ the patient underwent BR achieving a CR. At this time, BR had demonstrated to be an active regimen for indolent relapsed/refractory NHL (Rummel et al. 2005) and for patients with relapsed/refractory DLBCL (Vacirca et al. 2014), leading to a ORR of nearly 46% with a median duration of response of 17.3 months with a favorable toxicity profile. Therefore it could represent an interesting treatment option for patients who are inelegible for a platinum based chemotherapy and consolidation with autologous stem cell transplantation.

In recent years, the efficacy of many novel molecules has been evaluated in several international clinical trials, but only few of them proved to be efficient. One of them was Everolimus, a mTOR inhibitor, used as a single-agent in elderly, heavily pretreated patients affected by aggressive lymphomas (Witzig et al. 2011). Results were promising and an ORR of 30% and time to relapse of nearly 6 months were observed. Similar results were obtained using lenalidomide, an immunomodulating agents, as single-agent in 73 relapsed/refractory DLBCL patients (Wiernik et al. 2008). In comparison with the results of these trials, BR had the best long-term results with a almost double the time to relapse and a more favourable toxicity profile.

To our knowledge, this is the first report regarding the use of BR as salvage therapy in relapsed/refractory PBL. Despite the aggressive clinical course, BR in fourth line treatment was able to induce a CR and to maintain it for a year – a comparable result to the more aggressive R-CHOP administered in first line. Moreover, despite the advanced age of the patient, no important hematologic and extrahematologic toxicities occurred.

In conclusion, BR seems to be an effective salvage immunochemotherapy regimen for refractory PBL not eligible for a platin containing salvage chemotherapy and ASCT.

### Consent

Written informed consent was obtained from the patient for the publication of this report and any accompanying images.

## Authors’ information

Patrizia Mondello, MD, graduated with mark 110/110 cum laude in Medicine and Surgery at the University of Messina (Italy) in 2009 defending an experimental thesis entitled “Primary Lymphoma of the central nervous system” and is currently attending the fourth year of the post-graduate specialization in Oncology. She pursues different cancer specializations, focusing on the hematoncology field. She has received many awards and spent long periods in Germany and the USA, furthering her medical and language knowledge. She is currently spending a year at the Memorial Sloan Kettering Cancer Center in New York working in the laboratory of Dr. Anas Younes as research fellow and furthering her knowledge of translational research in the lymphomas field. She is also co-author of scientific papers published in national and international peer-review journals. Her main fields of research focus on pathophysiology in onco- and hematology fields and translational research.

Michael Mian graduated in Medicine at the University of Innsbruck in 2004. In 2005 he worked as resident at the University of Salzburg. In 2009 he specialized in hematology at the University of Verona. From 2009 to 2010 he was a research fellow at the the Institute of Oncology Research of the Istituto Oncologico della Svizzera Italiana for 1.5 years. During this fellowship he developed methods to interpret single nucleotid polimorphism array data together with clincal data. Since then, he has been working as a physician at the General Hospital of Bolzano. In collaboration with the International Extranodal Lymphoma Study group he provided new insights into the clinical behaviour of rare extranodal lymphoid malignancies.

Carmela Arrigo, lab manager of Stem of “High dose chemotherapy and Stem cell transplantation department” at Universitary Hospital “G. Martino” in Messina. She graduated in Natural Science in 1975 and in 1986 in Medical Biology at the University of Messina. She specialized in “Medical Genetics” in 1993 at the University of Catania. She is also co-author of scientific papers published in national and international peer-review journals.

Vincenzo Pitini, MD and Chief of “High dose chemotherapy and Stem cell transplantation department” at Universitary Hospital “G. Martino” in Messina. He graduated in Medicine and Surgery in 1977 with 110/110 cum laude. After graduation, he specialized in Renal, haematological diseases, and metabolic disorders in 1980. He specialized in Oncology in 1983. Since August 1980, he has been University Researcher at the Institute of Oncology at the University of Messina, and is still in service at the Department of Human Pathology. Since 1987 he has obtained the qualification of Aid. Since the academic year 1990/91 he has also given lesson cycles on the use of Molecular Biology in Oncology. Since 1995, he has developed the procedures for the collection and subsequent reinfusion of circulating stem cells in the high-dose antiblastic therapy, thereby contributing to the accreditation of the Division of Medical Oncology at the Italian Group for Bone Marrow Transplantation (GITMO) CIC 669. He also attended an updating course in Oncohematology at the University of Texas M.D. Anderson Cancer Center in Houston, Texas (USA). He is also author of scientific papers published on national and international peer-review journals focusing on hematology and oncology fields.

## Abbreviations

PBL: Primary bone lymphoma
DLBCL: Diffuse large B-cell lymphomas
ASCT: Autologous stem cell transplantation
BR: Bendamustine plus rituximab
CT: Computed tomography
PET: Positron emission tomography
FDG: Fluorodeoxyglucose
CD: Cluster of differentiation
R-CHOP: Rituximab, cyclophosphomide, doxorubicin, vincristine and prednisone
PR: Partial remission
90Y-IY: 90Y-ibritumomab tiuxetan
CR: Complete response
RT: Radiotherapy.
